# Scientific Basis of Mind-Body Interventions

**DOI:** 10.1155/2014/706892

**Published:** 2014-03-05

**Authors:** Hector Tsang, Cecilia L. W. Chan, Kevin Chen, William Chi-shing Cho, Myeong Soo Lee

**Affiliations:** ^1^Neuropsychiatric Rehabilitation Laboratory, Department of Rehabilitation Sciences, The Hong Kong Polytechnic University, Hong Kong; ^2^Centre on Behavioral Health, Department of Social Work and Social Administration, The University of Hong Kong, Hong Kong; ^3^Center for Integrative Medicine, The University of Maryland School of Medicine, USA; ^4^Department of Clinical Oncology, Queen Elizabeth Hospital, Hong Kong; ^5^Medical Research Division, Korea Institute of Oriental Medicine, Daejeon 305-811, Republic of Korea

Mind-body exercise, a form of exercise that combines body movements with mental focus, has a long and winding history around the world. In the two ancient civilizations, India and China, Yoga and Health Qigong/Tai Chi have, respectively, been practiced for thousands of years among healthy individuals or those with a health condition for wellness or therapeutic purpose. In modern times, progressive muscle relaxation, visualization, and Pilates are becoming more and more popular in the western world for health and fitness.

Although different types of mind-body exercises have been practiced by people from different cultures and clinical effects have been observed for centuries, these interventions have been criticized by researchers and practitioners to be lacking in scientific support in two aspects. First, many review articles [1, 2] (Ng and Tsang, 2009; Wang and Chan et al.) reveal that most clinical studies on these mind-body interventions are not well controlled and designed. Many do not even have a comparison or control group. These have posed a lot of difficulties on the generalizability of the results. Second, even effects are well documented. The biological or psychosocial mechanisms underlying the effects are largely unknown [3, 4].

My neuropsychiatric rehabilitation research team at The Hong Kong Polytechnic University has been active doing research in providing scientific evidence to Chinese mind-body exercises for the past decade. I am glad to receive many requests for reprints after the research articles are published. Some even requested for access to the video so that they may replicate the studies or apply these mindfulness exercises in their clinical settings. It is an excitement when I was asked by the editorial office to edit a special issue devoted to the scientific basis of mind-body exercises. This special issue in fact is to fill the knowledge gap that may help promote these activities for clinical practice and gradually attain its mainstream status in modern medicine.

In this special issue, we are happy to present a total of 7 original research papers to augment the evidence base for mind-body interventions. It is impressive to see that mind-body/mindfulness interventions have been applied to different groups of clients including frail elders, patients with major depressive order, shoulder pain, chronic fatigue, acute respiratory infection, and patients in intensive care unit. Articles submitted to this special issue provide further evidence on the clinical effectiveness in various aspects. H. W. H. Tsang and his team showed that the newly developed adapted mind-body exercise for frail elders improved their thinking operations. EEG data from A. S. Chan et al. and P.-C. Lo and C.-H. Chang showed that Chan-based mind-body intervention helped induce positive mood and calm state mind. J. S. M. Chan et al. reported that Wu Xing Ping Heng Gong reduced total fatigue score among chronic fatigue patents. A. L. Baldwin and colleagues showed that energy healing was as effective as manual manipulation physical therapy in treating patients with shoulder pain problem. A. M. Chiasson et al. realized that patients in intensive care unit found harp music effective in helping them reduce perceived pain. Finally, the mindfulness based stress reduction program by A. Zgierska et al. has been demonstrated to produce improvement in psychosocial functioning among patients with acute respiratory infection.

I hope that the results reported in this special issue will further spark further enthusiasm on scientific research for mind-body interventions in the future so that more clients will benefit providing evidence, clinical implications, and inspiration to clinicians, researchers, and patients.

*Hector Tsang* *Hector Tsang* *Cecilia L.W. Chan* *Cecilia L.W. Chan* *Kevin Chen* *Kevin Chen* *William Chi-shing Cho* *William Chi-shing Cho* *Myeong Soo Lee* *Myeong Soo Lee*
